# Carbon Nanotubes-Based Assays for Cancer Detection and Screening

**DOI:** 10.3390/pharmaceutics14040781

**Published:** 2022-04-03

**Authors:** Cristina Bura, Teodora Mocan, Cristiana Grapa, Lucian Mocan

**Affiliations:** 1 Nanomedicine Department, Regional Institute of Gastroenterology and Hepatology ‘’Octavian Fodor’’, 400008 Cluj-Napoca, Romania; gastromm@gmail.com (C.B.); teodora.mocan@umfcluj.ro (T.M.); cristianagrapa@yahoo.com (C.G.); 2 Department of Physiology, University of Medicine and Pharmacy, “Iuliu Hatieganu”, 400008 Cluj-Napoca, Romania; 3 Department of Surgery, University of Medicine and Pharmacy, “Iuliu Hatieganu”, 400008 Cluj-Napoca, Romania

**Keywords:** carbon nanotubes, cancer, diagnosis, markers

## Abstract

Carbon nanotubes (CNTs) were considered a potential cargo for cancer therapy and diagnosis following researchers’ shared goal of finding a new delivery system to enhance the pharmacological performance of the administered drugs. To date, several excellent reviews have focused on the role of CNTs as drug delivery systems, although there is currently no existing study that gathers all the advances in research-connected carbon nanotubes-based assay development for the early detection of cancer. In this review article, we will focus on the emerging role of CNTs as anticancer detection agents.

## 1. Introduction

Being an academically integrative field of research, nanotechnology, which links the involvement of physics, chemistry, biology, and medicine, could be a key factor in the attainment of an earlier detection, proper diagnosis, and more effective treatment that suit each patient’s individual needs. The new physical properties arising from the nanoscale phenomena are considered to be some of the most important foundations for nanotechnology [1,2]. The processes of diagnosis and treatment possess the potential of being radically transformed as a result of nanoparticles (such as single-walled carbon nanotubes [SWCNTs]), which, with the size of many orders of magnitude smaller than that of human cells, present remarkable interactions with biomolecules both on the surface and inside the cell. Near the end of the twentieth century, following great progress involving the discovery of multi-walled carbon nanotubes (MWCNTs) as well as the synthesis of SWCNTs, the scientific world was introduced to a whole new area of possibilities regarding research on the nanoscale [3,4,5]. As a result, in the past decade, astounding growth in the field of molecular imaging can be noted: ‘‘the visualization, characterization and measurement of biological processes at the molecular and cellular levels in humans and other living systems’’ [6,7,8,9]. Molecular magnetic resonance imaging (MRI), magnetic resonance spectroscopy (MRS), optical bioluminescence, optical fluorescence, targeted ultrasound, single-photon emission computed tomography (SPECT), and positron emission tomography (PET) are customarily the current standard molecular imagining modalities. Factors such as persistent advancement and generally easier access to scanners conductive of small animal imaging studies (demonstrated as capable of determining a similar in vivo imaging capability in mice, primates, and humans) can facilitate a stable and fluent transfer of knowledge and molecular measurements between species, potentially resulting in an easier process of clinical translation [10,11].

Carbon nanotubes (CNTs) were considered a potential cargo for cancer therapy and diagnosis following researchers’ shared goal of finding a new delivery system to enhance the pharmacological performance of the administered drugs [12,13,14,15,16]. Cylindrical in shape, CNTs are known to belong to the fullerene family of carbon allotropes [17]. At the current time, two types of carbon nanotubes are used for biomedical applications: single-walled carbon nanotubes-a single layer of grapheme with a diameter of 1.2–2 nm and a thermal conductivity of 6000 W/m*K, these can be easily twisted but purity is low.

Multi-walled carbon nanotubes are formed by multiple grapheme layers, they have a diameter of 5–100 nm, and high purity. They are suitable in biomedical applications since they easily can be synthesized and characterized and can be easily twisted. Their thermal conductivity is 3000 W/m*K.

To date, several excellent reviews have focused on the role of CNTs as drug delivery systems, although there is currently no existing study that gathers all the advances in research-connected carbon nanotubes-based assay development for the early detection of cancer. In this review article, we will focus on the emerging role of CNTs as anticancer detection agents.

**Figure 1:**
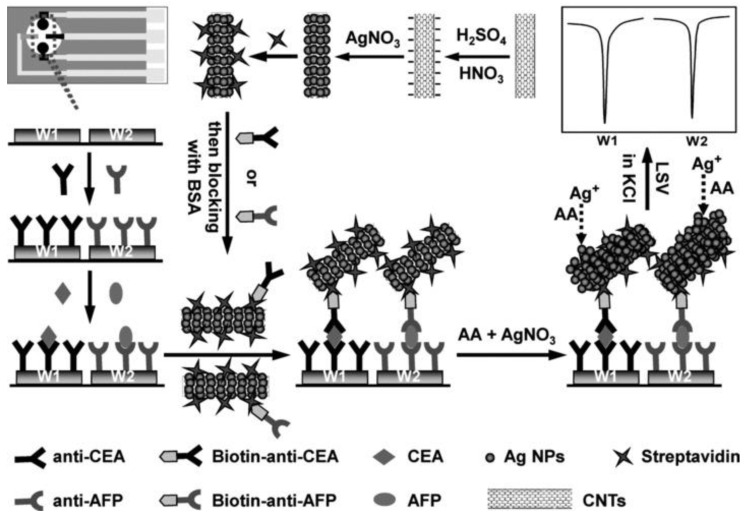
Schematic representation of preparation of immunosensor array and trace tag, and detection strategy by linear-sweep stripping voltammetric analysis of Ag NPs on the immunosensor surface. Reproduced with permission from [18], Wiley, 2011.

It is a known fact that rapid identification of any tumor is an important step that can make the difference between curative and palliative treatment. Biomarker levels in either blood or tissue can be useful for diagnosis, follow-up, and prognosis for many types of cancers, and a vast number of immunoassays have been employed for tumor marker identification. Based on these facts, a team of researchers developed a nanoprobe composed of carbon nanotubes enriched with silver nanoparticles and functionalized with streptavidin in order to obtain an immunoassay for the detection of α-fetoprotein or carcinoembryonic antigen. The immunosensor used screen-printer carbon electrodes which could covalently bind the antibodies; it proved to have a high detection precision of 0.061 pg mL^−1^ and 0.093, respectively. The assay was also able to overcome some barriers such as the generation of cross talk or the need for deoxygenation that are common with electrochemical immunoassays, and thus providing a new, improved and highly sensitive detection method for tumor markers (Figure 1) [18].

Another team also tried to develop a biomarker detection method based on nanoprobes, to amplify the detection signal. They used MWCNT with horseradish peroxidase (HRP) multilayers and conjugated with alfa-fetoprotein (AFP) antibodies. HRP was bound to the nanocarrier using an electrostatic layer by layer method; this led to SWNT being negatively charged, so positively-charged poly(dimethyldiallylammonium chloride) (PDDA) was also added; the two combined led to a great signal intensification. The final step was employing luminol–H2O2-HRP-bromophenol blue (BPB), achieving a chemiluminescence detection system for AFP in human serum samples. The results obtained were compared to the ELISA method in for the characterization of the applicability of the method created. The assembly could reach a detection limit of 8.0 pg/mL, lower than the ELISA standard method. The system provides promising new strategies for biomarker detection using nanomaterials [19].

Electrical immunosensors for tumor biomarkers hold great promise in improving point-of-care systems and early detection of cancer; they represent simple, sensitive, and feasible procedures that could help save millions of lives [20,21,22]. An electrical immunosensor for prostate antigen (PSA) detection was developed by Yang et al. using MWNTs, gold nanoparticles and as signal molecules–a secondary antibody (Ab2) and 6-ferrocenyl hexanethiol (Fc) labels. The detection limit for the label created was of 5.4 pg·mL^−1^ in serum samples and a linear range from 10 pg.mL^−1^ to 100 ng.mL^−1^. The approach is based on the multiple signal amplification capabilities of both the carbon and gold nanotubes, generating encouraging results. The authors point out that this type of immunosensor could also be employed for the detection of other tumor markers [23].

To meet the high demand for signal amplification methods, researchers have continuously developed new and improved technologies, including DNA rolling circle amplification (RCA). The DNA tags are conjugated with antibodies that enhance the recognition of the antibody-antigen complex, while the RCA can replicate the DNA template up to thousands of times in a couple of minutes. Using this system, a team established a MWCNT rolling circle amplification procedure to detect AFP and PSA levels in different samples. DNA primers and Streptavidin were added to the nanocomplex. The detection limit for the chemiluminescent complex was 5 fg mL^−1^ for AFP and 10 fg mL^−1^ for PSA; compared to the standard ELISA procedure, this technique was 4000 times more sensitive. It also performed outstandingly well in for detection of biomarkers in biological fluids, therefore representing great promise for the development of innovative and superior biosensors [24].

The carbohydrate antigen CA 15-3 is a commonly used biomarker for breast cancer diagnosis and prognosis evaluation. Sadhasivam et al. developed a biosensing nanoprobe for the detection of CA 15-3, using polymerase chain reaction (PCR). The nanotubes were collected on hydrofluoric acid (HF) to avoid non-specific binding and then treated with silicon water; the resulted nanoprobe was chemically treated and functionalized with CA 15-3 antibodies. The next step was represented by the non-specific binding of biotinylated DNA; streptavidin was used for bonding the secondary biotinylated antibodies. The attached DNA was additionally magnified by PCR using specific primers. The final result was the development of an antibody–antigen–antibody–DNA conjugate. Electron microscopy, spectrometry and gel electrophoresis were used for the characterization of the nanoprobe. The biosensor proposed showed a lower limit of detection of 0.001–0.01 U/mL using PCR. The team thus developed a sensitive sensor for CA 15-3 identification [25].

An interesting approach for biosensor development was took by a team of researchers [26] when they used vertically aligned carbon nanotubes (VACNTs) for the development of an electrical spectroscopy device. Its purpose was to diagnose cancer metastasis based on individual cell membrane impedance with single-cell resolution. They managed to electrically couple the CNT beams with the cell membrane, in order to acquire information such as electronic resonance, ionic permeability and measure cell resonance peaks at a single cell level. Their premise is represented by the fact that a high number of biological alterations in a cell are determined by the electrochemical changes in the membrane The mechanism could thus be used for the detection of circulating cancer cells, or tumor staging/grading, and it would only need a few cells, in comparison to the abundance of tissue that pathologists usually need to place a diagnosis. Metastatic colon and breast cancer cells were successfully diagnosed using this method. CNT spectroscopy could therefore represent a new method for characterization of the electrical changes that occur during cancerous transformation, improve diagnosis and aid with clinical decisions regarding treatment.

## 2. Results and Discussion

### 2.1. Breast Cancer

At the current time intensive research is conducted in the field of breast cancer diagnosis using carbon nanotubes [27,28,29,30,31,32,33].

Single-walled carbon nanotubes (SWNTs) were used by Welsher et al. [34] for their proprieties as near-infrared (NIR) fluorescent markers in order to detect breast cancer cells. They were first functionalized with polyethyleneglycol, to increase their hydrophilicity; secondly, they were conjugated with Rituximab, a monoclonal antibody that targets the CD20 cell surface protein, which is highly expressed on B cells, and Transtuzumab (Herceptin), another monoclonal antibody that binds to the HER2 receptors of breast cancer cells. The nanoconjugates selectively bound the to breast cancer cells lines, while the SWNTs photoluminescence provided a highly sensitive imaging method. An advantage provided by the use of carbon nanotubes was the by-passing of cellular autofluorescence, thus providing an advanced technique of detecting even the cells with the lowest expression of cell surface receptors.

Another team of researchers used SWNTs capacity to absorb NIR light in order to selectively destroy breast cancer cells through photothermal ablation; they also exploited their strong Raman signaling for the detection of cancer cells, proving a dual role for the nanocomposite [35]. Breast cancer cell lines expressing the HER2 receptor were targeted with SWNTs conjugated with anti-HER2 IgY antibody; the mixture was detected using a Raman spectrometer, which showed high signal collection of the nanocomplex treated cells, compared to controls. Next, Xiao et al. proved that NIR irradiation of the treated cell lines leads to extensive cell death; the method applied did not require nanoparticle internalization, which is unusual compared to other studies. Another particularity of the study is represented by the use of IgY avian antibodies, instead of IgG mammalian ones; research using these types of antibodies is scarce, but the author’s results are promising, since IgY offer advantages such as less interference with other immunological assays and availability in large quantities. The present study thus reveals the dual role of SWCNTs for selective imaging and therapy of HER2 expressing cells.

A nanoplatform was developed by Wang et al. [36] using multi walled carbon nanotubes (MWNT) with iron oxide, functionalized with polyethylenimine (PEI) and PEG, for magnetic targeting and magnetic resonance imaging (MRI). They further bonded human telomerase reverse transcriptase (hTERT) siRNA and administered the nanocomplex to MCF-7 breast cell lines; this drug delivery system led to significant tumor inhibition, by gene silencing; it also proved useful for magnetic targeting, MRI imaging and photothermal tumor cell inhibition. The study adds to the multitude of new research in the field of tumor theranostics using nanomedicine.

Cancer stem cells (CSC) have proved to be an important part of any tumor, including breast cancer, due to their ability to initiate and maintain cancer cell replication and lead to poor therapy response. In a study conducted by Al Faraj [37], a drug delivery system (DDS) composed of SWCNTs conjugated with CD44 antibodies (due to the fact that CD44 is largely present on the surface of CSC), and different imaging tracers, such as superparamagnetic iron-oxide nanoparticles, gallium-67 or NIR fluorophores were administered to murine models of breast cancer. The administration of the nanoprobe led to enhanced accumulation at the tumor site and increased imaging efficacy. The authors point out that biocompatibility studies need to be carried out before implementing this type of DDS, but the results offer hope for better targeting and imaging of cancer cells.

The use of nanomaterials as contrast agents for photothermal therapy (PTT) has been intensively studied in the last past years; research has proved that carbon nanotubes are a convenient choice for this type of application. Based on this, a team of researchers [38] developed a nanoprobe composed of single wall carbon nanohorns (SWNH) modified with poly(maleic anhydride-alt-1-octadecene-poly(ethylene glycol)) (C 18 PMH-PEG), which led to a highly biocompatible and stable probe that could serve as photoacoustic agent for visualization of breast cancer cells, in vitro and in vivo. Photoacoustic imaging (PAI) showed accumulation of the nanocomplex in the tumor vessels, thus clearly delineating the tumor, and PTT therapy led to effective tumor inhibition with minimal recurrence. No noticeable side effects in mice were reported during the study. The study also revealed that the maximum accumulation of the nanocomplex was after 24 h, which is considered also the optimal time for PTT therapy. Hence, a biocompatible theranostic agent is proposed for PAI and PTT therapy of breast cancer cells.

Another successful attempt at using SWNT as molecular carriers for DDS in cancer imaging and therapy was created by combining the nanoparticles with integrin αvβ3-mAb (monoclonal antibody) as a targeting marker [39]. The integrin was chosen because of numerous studies that linked it to tumor progression, metastasis and angiogenesis, and antagonists of this integrin, such as monoclonal antibodies, have demonstrated good results for tumor inhibition. The team thus developed a nanoprobe with high biocompatibility, non-toxic, that could specifically target breast cancer cells in vitro. They compared cellular lines that had high integrin expression with lines that had low αvβ3 expression and concluded that for cells with the highest expression the nanoprobe had the greatest targeting efficiency. This proves the importance of using cellular targets in order to improve therapeutic efficacy.

As stated, attaching nanomaterials to different targeting agents is an important step in improving cancer diagnosis and treatment. Lately, epithelial cell adhesion molecules (EpCAMs) have emerged as promising targeting molecules for different types of cancers, seeing as they are overexpressed in many epithelial tumors. Antibodies that target EpCAM were tied to carbon nanotubes in an experiment developed by Nima et al. [40] in order to better visualize breast cancer cells. The nanocomplex formed by covalently bonding SWNTs with anti-EpCAM was administered to MCF-7 cell lines and to normal skin fibroblast which did not express EpCAM. Imaging of the nanoprobes was performed using Raman spectroscopy; the covalent bond between EpCAM and SWNT was confirmed through transmission electron microscopy. Raman signal of the MCF-7 treated cells highly increased after only 30 min of incubation and reached a maximum after 12 h; furthermore, no Raman signal was detected from the fibroblast cells. The authors concluded that this nanoprobe can successfully be used for specific identification of breast cancer cells.

**Figure 2:**
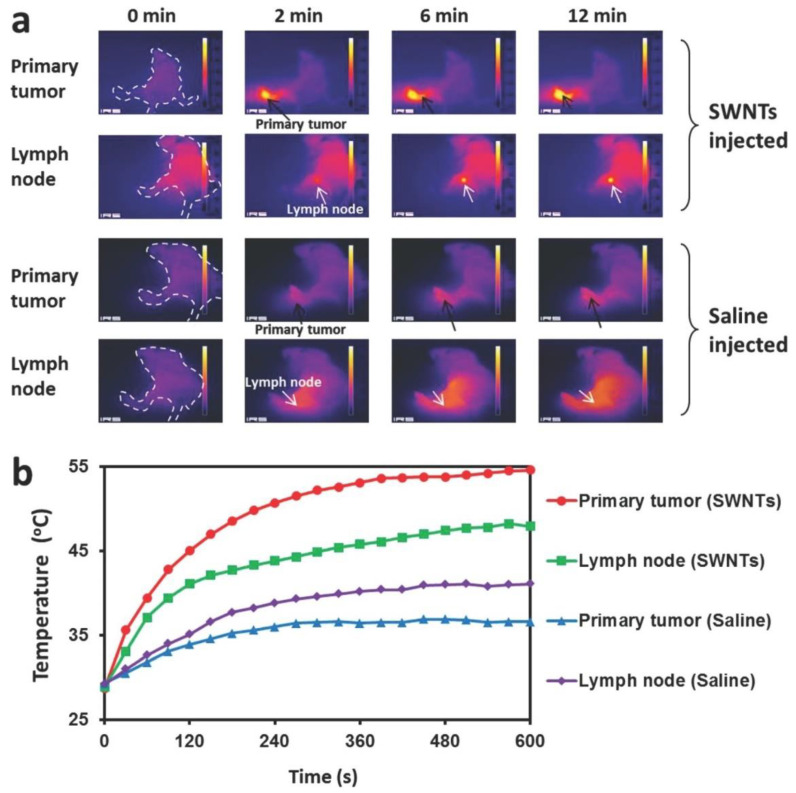
In-vivo photothermal heating. (**a**) IR thermal images of 4T1 tumor-bearing mice with the primary tumor and the popliteal lymph node separately exposed to the NIR laser at different power densities of 0.5 W cm^−2^ and 0.8 W cm^−2^, respectively. The heating experiment was started 90 min after injection of SWCNT–PEG or PBS into the primary tumor. The primary tumors and lymph nodes are pointed out by black and white arrows, respectively. The dose of SWCNTs was ca. 0.2 mg kg^−1^. (**b**) Temperature elevation on the primary tumor and the lymph node monitored by an IR thermal camera during laser irradiation as shown in (**a**). SWCNT injection resulted in rapid temperature rises of both the primary tumor and the popliteal sentinel lymph node. Reprinted with permission from [41], Wiley, 2014.

Seeing as tumor metastasis is responsible for a representative percent of cancer deaths, Liang and his team [41] developed for the first time a nanoprobe for image-guided photothermal therapy of a primary tumor and its metastasis in sentinel lymph nodes, using a mouse tumor model. Based on the unique propriety of SWNTs emitting fluorescence in the NIR-II region (1000–1700 nm), PTT was applied to the primary tumor; in vivo imaging revealed the migration of the nanoprobe through the lymphatic circulation into a sentinel lymph node, thus offering the opportunity to efficiently guide PTT to the metastasis site. The survival of the treated mouse models was astonishingly prolonged compared to mice which were treated with only primary tumor elimination (Figure 2). The study therefore shows a promising new diagnostic and therapeutic approach for metastatic cancers.

The ability to track carbon nanotubes in different biological environments has been extensively studied, with promising results so far. SWNTs have become an attractive tool for this purpose, as they present with a hollow internal space that can be loaded with either contrast agents or bioactive agents and a surface that can be functionalized to obtain a better targeting agent. Based on this premise, Al Faraj [42] conducted an experiment using iron-tagged SWNTs administered to a murine model of breast cancer in order to test its capability as a DDS. The carbon nanotubes were loaded with iron oxide to enhance their magnetic effect and were also functionalized with a CD105 monoclonal antibody for active targeting. In vivo imaging was performed using an MRI protocol, showing high accumulation of the nanoprobe at the tumor site. The biocompatibility of the probe was also tested through cell viability assays, free oxygen radicals detection or detection of changes in the mitochondrial membrane potential. The study showed the highest accumulation for the conjugated nanoprobes, with good biocompatibility.

Another successful attempt in cancer theranostics was provided by a team of researchers who used SWNTs functionalized with doxorubicin, modified with hyaluronic acid and loaded with gadolinium which was delivered to MCF-7 breast cancer cells [43]. The system led to enhanced drug delivery and tumor cell apoptosis, improved contrast for MRI imaging and good biocompatibility. This multifunctional nanoprobe showed great potential for cancer theranostics, proving once again that carbon nanotubes have significant advantages and should be further tested for clinical implementation.

Identification of sentinel lymph nodes (SLN) for breast cancer patients is an important step in cancer staging, so a feasible rapid method for identification could improve survival. Koo et al. [44] used SWNTs as contrast agents for photoacoustic imaging (PA) of breast cancer cells. They added indocyanine green (ICG) to the nanoparticle, in order to improve PA sensitivity for sentinel lymph node detection. In vivo imaging showed high accumulation of the SWNTs-ICG at the tumor site; the authors also used this type of naboprobe for a better visualization of the bladder, with good results. The current study thus proposed a new non-invasive imaging method for SLN and vesicoureteral reflux detection.

### 2.2. Lung Cancer

Seeing as lung cancer is one of the commonest forms of cancers that affects millions of patients, researchers have tried to employ nanotechnology for its diagnosis and treatment. In one study, a team of researchers proposed an innovative new diagnostic tool based on breath samples belonging to lung cancer patients [45]. They developed a network from SWNT coated with organic films and functionalized with lung cancer biomarkers. The network showed great results for cancer detection, when compared to healthy controls. The device is easy to develop, inexpensive and if it could be implemented, would help in the early diagnosis for this category of patients and improve their survival chance.

Another team also employed the use of SWNT for in vivo lung cancer multimodal imaging [46]. Gadolinium and copper were loaded onto the carbon nanotubes by sonication in order to ensure imaging contrast and then the nanoprobes were injected into tumor free athymic mice. The probe was stable for up to 48 h, according to PET imaging. Seeing as there was significant accumulation of the probe in the lungs, the authors proposed that this type of device would be useful for various lung diseases, including cancer. T1-MRI imaging was also performed, thus providing a promising new imaging guided DDS, using the hybrid PET/MRI system. This type of system could be useful in many types of cancers for determining the optimal therapeutic doses or visualizing distribution for various chemotherapy agents.

### 2.3. Brain Cancer

**Figure 3:**
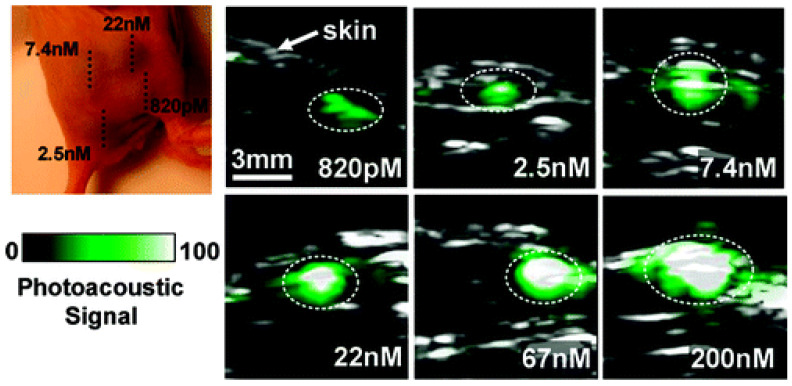
Photoacoustic detection of SWNT-ICG in living mice. Mice were injected subcutaneously with SWNT-ICG at concentrations of 0.82–200 nM. The images represent ultrasound (gray) and photoacoustic (green) vertical slices through the subcutaneous injections (dotted black line). The skin is visualized in the ultrasound images, while the photoacoustic images show the SWNT-ICG distribution. The white dotted lines on the images illustrate the approximate edges of each inclusion. The photoacoustic signal from each inclusion was calculated using 3D regions of interest and the “background” represents the endogenous signal measured from tissues. The error bars represent standard error (n) 3 mice). Linear regression (R2) 0.97) of the photoacoustic signal curve estimates that a concentration of 170 pM of SWNT-ICG will give the equivalent background signal of tissues. Reprinted with permission from [48], American Chemical Society, 2010.

As stated, research on carbon nanotubes as imaging contrast agents for photoacoustic imaging has gained a lot of ground. De La Zerda et al. proved that after iv administration of targeted SWNT to tumor bearing mice there was an increase in PA signal up to eight times higher compared to non-targeted SWNT. After surgically removing the tumors, Raman spectroscopy was also used to verify their findings [47]. Continuing their research, the same team used indocyanine green attached to SWNT and administered the complex to glioblastoma bearing mice (Figure 3). They further conjugated the probe with cyclic Arg-Gly-Asp (RGD), which are able to target integrins involved in tumor angiogenesis. The new contrast agent developed led to an increase in PA imaging contrast of 300 times greater than previous reports, achieving subnanomolar sensitivity. There is no other imaging agent reported to achieve this kind of sensitivity, so this dye-enhanced nanoprobe could substantially improve imaging for brain tumors [48].

In accordance with previous mentioned studies, another team of researchers developed SWNT conjugated with integrin v3 antibodies, for PA in vivo and in vitro imaging of glioblastoma tumors in mice [49]. They chose this particular antibody because of integrin v3 overexpression in U87 glioblastoma tumors. In vivo toxicity tests were applied which proved that the nanocomplex used was biocompatible, with minimal cellular toxicity. The authors also characterized elimination of nanoprobes, showing that the complex is not retained in the reticuloendothelial system, but rapidly eliminated through the renal system. Clinical translation of these findings is critical, as it could lead to significant improvements in the field of cancer therapy.

### 2.4. Ovarian Cancer

Gynecologic diseases are a global health problem and novel technologies toward early diagnostics [50,51,52] and improvement in cancer therapy are required [53].

Regarding ovarian cancer, Ghosh et al. [54] developed an M13 bacteriophage stabilized SWNT complex that had the ability to selectively aim the secreted protein acidic and rich in cysteine system (SPARC) in mouse models of this particular type of tumor. The team also examined the nanoprobe’s in vitro ability to target OVCAR8 ovarian cancer cells. Fluorescence imaging showed high tumor uptake and improved image guiding. The probe developed also showed improved imaging for deep tumors, reporting the highest depth of detection so far.

### 2.5. Pancreatic Cancer

Taking into consideration the fact that the epidermal growth factor receptor (EGF) is overexpressed in pancreatic cancer, Karkamar et al. [55] functionalized single wall carbon nanotubes with EGF and administered them to pancreatic cancer cells (PANC-1). Raman spectroscopy and enzyme-linked immunosorbent assay (ELISA) were used for characterization of the nanoprobe’s kinetics. The study revealed large accumulations of the functionalized nanoparticles near or inside the tumor cells, proposing an important role for EGF conjugation, seeing as laser irradiation of the PANC-1 cells containing the nanoprobes led to significant apoptosis, compared to non-targeted probes. This process could therefore be utilized for specific targeting of pancreatic tumor cells.

### 2.6. Cervical Cancer

Functionalized dendrimers were covalently bound to MWCNTs in order to target KB cancer cells lines which overexpress the folic acid receptor (FAR). Fluorescein isothiocyanate and folic acid modified dendrimers were used as multifunctional dendrimers. The nanocomplex proved to be stable and biocompatible, and in vitro studies using confocal microscopy or flow cytometry showed that it could specifically target cancer cells. The authors propose that by simply adding the multifunctional dendrimers to the carbon nanotubes, a complex targeting system for FAR overexpressing cells, with minimal toxicity and potential therapeutic use can be developed [56].

Another team of researchers also used KB and Hella cell lines to administer different types of SWNT decorated with gold or silver nanoparticles, in the hopes of developing an optical probe for cancer imaging and treatment. The premise was that by attaching either silver or gold to the carbon nanotubes (SWNT-Au-PEG and SWNT-Ag-PEG, their surface enhanced Raman scattering (SERS) effect would be significantly amplified. In order to attain selective aiming of the cells, folic acid (FA) was also used, resulting in the following nanocomplex: SWNT-Au-PEG-FA. The study showed that the NIR absorption proprieties of the gold nano-shells can enhance Raman scattering, improving imaging of the targeted cells and augment their photothermal ablation. In contrast, KB cells which were treated with only SWNT-PEG-FA showed negligible Raman scattering, revealing the importance of the gold nanocomposite. PTT was performed, showing that the highest number of tumor cells killed were the ones treated with the gold functionalized nanocomposite. In conclusion, the authors point out that by combining carbon nanotubes with a metal such as gold, their performance in cancer theranostics would be even greater [57].

### 2.7. Prostate Cancer

Recent studies show promising results in the diagnosis of prostate cancer using carbon nanotubes [58,59].

**Figure 4:**
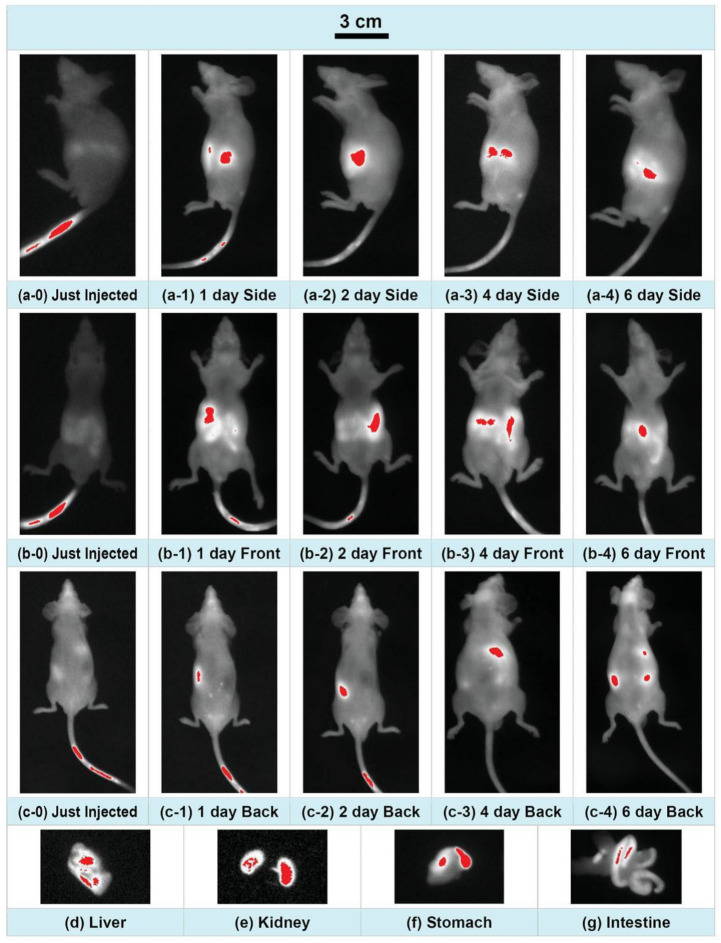
In vivo fluorescence images of CNT-QD IV injected into nude mice and imaged after circulation at various time intervals; (**a-0**–**a-4**) the images taken from the side of mice; (**b-0**–**b-4**) from the front of mice; (**c-0**–**c-4**) from the back of mice, and (**d**–**g**) organ images taken after having sacrificed the mice on the 6th day of post-injection. These pictures show prominent CNT-QD uptaking in the liver, kidney, stomach, and intestine. Reprint with permission from [60], Wiley, 2008.

Quantum dots (QD) conjugated carbon nanotubes are ideal for optical in vivo imaging of tumor bearing mice, as shown in the study conducted by Guo et al. [60]. After functionalizing them with PLGA and loading Paclitaxel, plasma mass spectrometry was used for in vivo imaging of the injected nanoprobes. Their viability and toxicity were assessed in vitro using prostate cancer cell lines, and the paclitaxel loading efficiency on the CN was assessed using liquid chromatography. To determine cell viability after paclitaxel administration, an MTT cell assay was used. The nanoprobe showed excellent antitumor efficacy against prostate cancer cells; mass spectrometry studies revealed uptake of the complex in the kidneys, liver and stomach after intravenous administration. The probe also showed optimal luminescence for non-invasive imaging (Figure 4). In conclusion, a novel optical probe for imaging and therapy in prostate cancer was developed using CN and quantum dots [60].

In another attempt to target prostate cancer cells, one study used MWCNT, polyethylenimine (PEI), fluorescein isothiocyanate (FITC) and prostate antigen monoclonal antibody (mAb) to form a complex nanoprobe. Tumor bearing mice were used for in vivo imaging, showing high tumor accumulation as well as growth inhibition. Flow cytometry and confocal microscopy combined for in vitro imaging proved that the nanoprobe can specifically target cells who overexpress the prostate antigen. Furthermore, in vivo and in vitro ultrasound imaging of the nanocomplex was performed, which showed that it could have good potential as a contrast agent. Cytotoxicity was tested using the WST-1 cell toxicity assay. Doxorubicin was also tested as for targeted delivery using this specific nanocomplex in mice, showing increased tumor apoptosis [61].

### 2.8. Gastric Cancer

Photoacoustic imaging using silica-coated gold nanorods added to the surface of MWCNT and conjugated with RGD for the characterization of gastric cancer cells was also performed. The gold nanorods were used for their ability to augment photoacoustic signal and improve the visualization of the tumor vessels. In vivo imaging of tumor bearing mice injected with the nanorods showed good tumor accumulation and enhanced photoacoustic signal. The explanation for this effect is given by the covalent interaction between the silica coated nanorods and the carboxyl terminal groups on the CNT. The authors also point out the potential use of the nanocomplex as a contrast agent. All in all, the probe proved to have good bioavailability and low toxicity, thus revealing its potential use in gastric cancer [62].

### 2.9. Liver Cancer

Magnetic resonance imaging has been established as an essential part of liver tumor characterization, and superparamagnetic iron oxide nanoparticles (SPIONs) have been extensively studied for their capabilities to enhance MRI signal. To this end, one study established that MWCNT combined with SPION, coated with PDDA and functionalized with a targeting agent, namely lactose-glycine adduct could successfully be used in order to improve MRI contrast for visualization of liver cancer cells. In vivo MRI imaging of tumor bearing mice was applied after intravenous administration of the nanoprobe, showing a 277% increase in contrast and high R2 relaxation time. The nanocomplex also proved to be biocompatible, non-toxic and have a high tolerance [63].

## 3. Conclusions and Future Prospects

Carbon nanotubes are extensively studied for their potential role in different biomedical applications (Table 1), and we have learned so far that usually their surface needs to be modified to make them soluble and increase their perspectives. Studies have given promising results for their use in different types of cancer, either for imaging or selective targeting of the tumors. Widespread efforts have been made to develop multifunctional nanoprobes that can be used as drug delivery systems, contrast agents, thermal agents etc., proving that nanotechnology could one day pave the way to an improved, less toxic cancer treatment. Research needs to continue this path, mainly to establish CNT’s pharmacokinetics and biodistribution, in order to successfully implement them in clinical trials.

**Scheme 1:**
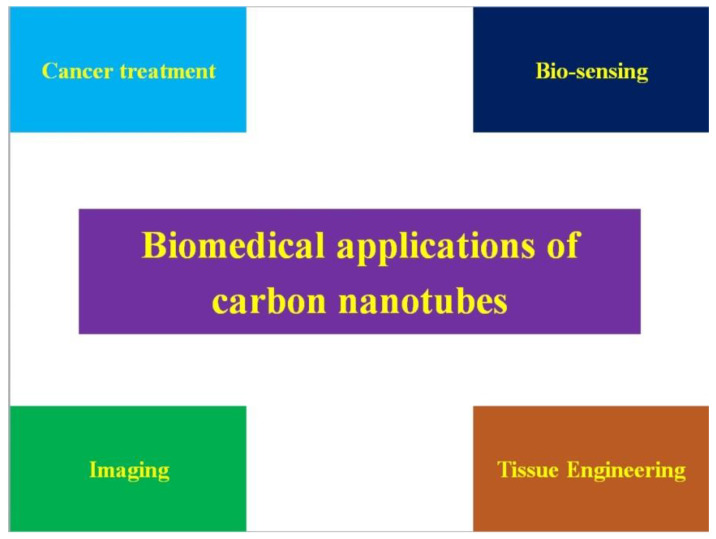
Graphic illustration of biomedical applications of carbon nanotubes.

The unique physical and chemical characteristics of CNTs make them one of the most promising agents for cancer detection and treatment (Scheme 1). The insight and perspective needed for CNT researchers to take their next step in cancer diagnosis using CNTs should focus on overcoming the major obstacles such as mitochondrial injury, inflammation, activation, DNA damage, adverse effects on cell performance, granulomas formation and fibrosis.

## Figures and Tables

**Table 1:** Cabon-based nanoparticles used in cancer diagnosis.

Nr. Crt.	Author	Type of Nanoparticle, Size	Size	Type of Cancer Detected	Method	Range of Detection/Tumor Size
	De la Zerda 2008 [47]	single-walled carbon nanotubes conjugated with cyclic ArgGly-Asp (RGD) peptides	Diameter:1–2 nm Length: 50–300 nm	Human Glioblastoma	Photoacoustic imaging	Ex vivo~150 mm^3^
	Xiao 2009 [35]	Carboxylated SWNTs with anti-HER2 chicken IgY antibody	Diameter:1.17 ± 0.28 nm Lenght 88.00 ± 43.68 nm,	Breast Cancer	Raman spectrometry	In Vitro~30,000 cells/cm^2^
	Robinson 2010 [64]	Short single-walled carbon nanotubes (SWNTs) functionalized by PEGylated phospholipids	Length~140 nm	Breast Cancer	Near infrared photoluminescence	In Vitro~2*10^6^ 4T1 cells
	Sai [19]	Multilayers enzyme-coated MWNTs	Diameter:20 nm Length: 3 µm	Liver Cancer marker	Chemiluminiscence	8 pg/mL
	Welsher2008 [34]	Single-walled carbon nanotubes (SWNTs)	Diameter:1.6 nm Length: 83 nm	Breast Cancer, Lymphoma	Near infrared photoluminescence	In Vitro
	Gosh, 2014 [54]	M13-stabilized single-walled carbon nanotubes (SWNTs)	Diameter:6 nm Length: 880 nm	Ovarian Cancer	Fluorescence Imaging	In Vitro~103 cells 1 mm^3^ tissue (at depths 9.7–18.2 nm)
	Karmakar, 2012 [55]	EGF functionalized SWNTs	Diameter:0.7–2.1 nm Length:0.2–3 µm	Pancreatic Cancer	Raman Spectroscopy	In Vitro
	Shi, 2009 [56]	Dendrimer functionalized MWNTs	Diameter:30–70 nm Length:100–2µm	Cervix Cancer	Fluorescence Imaging	In Vitro ~105
	Koo, 2012 [44]	ICG dyed single-walled carbon nanotubes (SWNTs-ICG)	N/A	Breast cancer (sentinel lymph nodes) Bladder cancer	Photoacoustic imaging	In vivo (rat)
	Al Faraj 2016 [37]	PEGylated SWCNT CD44 nanocarriers conjugated with superparamagnetic iron oxide nanoparticles (SPIONs) or radioactive Gallium-67	Diameter:1–2 nm Length:200 nm	Breast cancer	MRI, Single-photon emission computed tomography near-Infrared fluorescence imaging	In vivo (mice)
	Nima, 2013 [40]	Anti-EpCAM antibodies functionalized SWNTs	Diameter: 0.7 to 1.7 nm, Length:0.3 to 3 μm	Breast cancer	Raman spectroscopy	In vitro 2.5*10^3^/mL
	Liang, 2014 [41]	PEGylated SWNTs	N/A	Metastatic breast cancer	NIR-II fluorescent imaging	In vivo (mice)
	Wang, 2014 [62]	RGD-conjugated silica-coated gold nanorods on the surface of MWNTs	Diameter: 20~30 nm	Gastric cancer	Photoacoustic imaging	In vivo (mice) 5 mm^3^
	Antaris, 2013 [65]	SWCNTs exchanged into a biocompatible surfactant, C18-PMHmPEG	Diameter 0.7–1.1 nm	Breast Cancer	Near infrared photoluminescence	In vivo

## Data Availability

Not applicable.
